# Development of a probiotic low‐fat set yogurt containing concentrated sweet pepper extract

**DOI:** 10.1002/fsn3.4114

**Published:** 2024-03-15

**Authors:** Hossein Jooyandeh, Behrooz Alizadeh Behbahani

**Affiliations:** ^1^ Department of Food Science and Technology Agricultural Sciences and Natural Resources University of Khuzestan Mollasani Iran

**Keywords:** CSPE, hardness, microstructure, probiotic yogurt

## Abstract

Yogurt contains various health‐promoting components such as beneficial bacteria and bioactive peptides. However, plain yogurt is regarded as a poor source of phenolic compounds, vitamin C, and other substances that give a high antioxidant property to the product. Since the objective of this study was to evaluate the impact of the addition of different concentrated sweet pepper extracts (CSPE) to the yogurt milk formulation on some quality parameters of the probiotic set yogurt during 21 days of cold storage. For the production of probiotic yogurt samples, *Lactobacillus acidophilus* LA‐5 was used as probiotic bacteria. The functional set yogurts containing 5% of yellow, orange, or red CSPE were prepared and compared with probiotic control yogurt (without CSPE). With incorporation of CSPE, a functional yogurt with high viable cell counts of probiotics (>10^8^ cfu/g), appropriate acceptability (acceptance scores more than 7, i.e., good acceptability), and textural quality produced. Fortified CSPE yogurt displayed large flakes with irregular surface and more compact texture as compared to the control sample. Based on the results of the study, the yogurt sample having orange CSPE was selected as the best functional product. After 21 days of storage, orange CSPE had the highest hardness (173.33 mg), consistency (1183.33 mg/s), and probiotic counts (8.3 log cfu/g) than other yogurt samples.

## Graphical abstract

With the incorporation of concentrated sweet pepper extract (CSPE), a functional yogurt with high viable cell counts of probiotics (>10^8^ cfu/g), proper acceptability (acceptance scores more than 7, i.e., good acceptability), and appropriate textural quality produced. Fortified CSPE yogurt displayed large flakes with irregular surface and more compact texture as compared to the control sample. The yogurt sample having orange CSPE was selected as the best functional product.

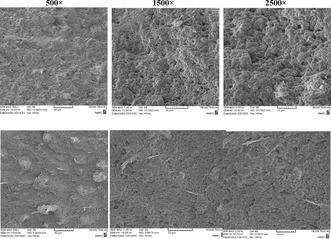

## 1 INTRODUCTION

People nowadays are more aware about their health and immune system, and therefore seek healthy food to support their intentions. Recently, there has been growing interest in assessing a diverse variety of functional foods that provide health benefits beyond their nutritional substances. Hence, the suggestion from the dairy division is more and more concentrating on the utilization of particular food additives that provide additional benefits to human health. Yogurt is one of the most popular fermented dairy products worldwide which its annual production is increasing progressively and has reached about 60.48 million tons in 2023 (Statista, 2023). Yogurt is a source of bioactive peptides which are developed throughout the process of fermentation. In addition, yogurt contains other health‐promoting components such as beneficial lactic acid bacteria (LAB) specifically probiotics that are beneficial to the function of human gut microbiota (Torabi et al., 2021). Probiotics are living microorganisms that confer a health advantage to the host when consumed in adequate amounts (Hill et al., 2014). Interestingly, a novel biological technique to eliminate the health threats of fungal toxins through adsorbing the toxins is the use of LAB and probiotic bacteria; particularly, in the areas where milk and dairy products are noticeably contaminated by these mycotoxins (Mosallaie et al., 2020). Furthermore, probiotic bacteria such as *Lactobacillus acidophilus* LA‐5 have the capability to decrease the serum cholesterol concentration, equilibrate and stabilize the intestinal microbiota, prompt the immune system, enhance lactose tolerance, and destroy tumor cells (Medellin‐Peña & Griffiths, 2009; Torabi et al., 2021). Therefore, the worldwide market of probiotics‐based products such as yogurt is assessed to reach 75 USD billion by 2025 (Latif et al., 2023). However, plain yogurt is regarded as a poor source of phenolic compounds, vitamin C, and other substances that give to the product's high antioxidant property. Consequently, the addition of natural functional ingredients rich in antioxidant substances such as fruits (Arslaner et al., 2021; Raikos et al., 2019) and vegetables (Jooyandeh et al., 2020, 2023) can boost the nutritional and nutraceutical benefits of the yogurt. Fortification with these bioactive constituents is one of the various methods to preclude the syndromes associated with dietary deficiencies. In addition to antioxidant activities, these compounds exert other beneficial health effects such as anticancer (Šaponjac et al., 2014), antiobesity (Maeda et al., 2013), anti‐inflammatory (Chen & Kang, 2013), and cardioprotective (Tsui et al., 2018) properties.

Bell or sweet pepper (*Capsicum annuum* L.) is a notable crop, not only because of its commercial values but also for its beneficial health effects, mainly due to its antioxidant compounds. In contrast to chili peppers, sweet peppers are larger fruits with little or no spicy taste. These vegetables are distributed to various areas of the world, including Europe, Middle East, and North America, and they are used freshly or cooked. Bell pepper species have a high amount of chlorophyll and carotenoid pigments, accountable for the green, orange, red, and yellow colors of the crops. These vegetables and their extracts are rich in phytochemical substances and differ in healthy factor substances depending on their color (Hong et al., 2020). Green bell pepper includes excessive amount of chlorophyll, while yellow and orange peppers have high levels of β‐carotene, zeaxanthin, and lutein, and red comprises carotenoid pigments, like capsorubin and capsanthin (Arimboor et al., 2015; Hong et al., 2020).

Recently, there has been rising attention to the application of natural food additives having health‐promoting compounds (Jooyandeh & Yademellat, 2017; Salehi, 2021). However, the incorporation of functional substances like plant extracts in foods may lead to decrease in product quality. Our previous work showed that the addition of three different colors (yellow, orange, and red) of concentrated sweet pepper extract (CSPE) increased antioxidant activity of the set yogurt (Jooyandeh et al., In Press). The amount of total phenolic compounds in control and yogurt samples containing orange, yellow, and red CSPE was determined as 129.56, 534.78, 399.22, and 423.78 μg/g, respectively. For antioxidant activity based on DPPH method, the values were recorded as 28.22, 68.11, 58.44, and 61.67%, respectively. Since the quality characteristics of probiotic yogurt largely depend on the number of probiotic bacteria, texture, internal microstructure, and organoleptic attributes, the objective of this study was to investigate the effect of CSPE on the mentioned features of functional low‐fat set yogurt.

## 2 MATERIALS AND METHODS

### 2.1 Materials

Low‐fat milk (1.5%) was obtained from Pegah Company (Shush, Iran). Sweet peppers with yellow, orange, and red colors were purchased from the local market. YF‐L811 yogurt culture and *L. acidophilus* LA‐5 (as probiotic bacteria) in lyophilized powder form (Christian Hansen Co, Denmark) were used for yogurt sample preparation. All chemicals and culture media used in this study were of analytical grade and usually were obtained from Merck (Germany).

### 2.2 Preparation of the concentrated sweet pepper extract

Sweet peppers with yellow, orange, and red colors were washed and crushed by a blender. After straining for pulp extraction through a couple of layers of cheesecloth, the total soluble solids of juices were adjusted up to 30% T.S. by a rotary evaporator at 60°C. The prepared CSPEs were made and used in the yogurt formulation on the day of sample preparation.

### 2.3 Preparation of yogurt samples

The set‐style yogurt samples were produced on a laboratory scale according to the method described by Yademellat et al. (2017) with some modifications. For yogurt production, the milk was heated at 90°C for 10 min and subsequently, the temperature was reduced to 65°C. After the addition of CSPEs, the milk temperature was adjusted to 44–45°C. According to the manufacturer's instructions, to obtain at least 10^8^ cfu of starter cultures or probiotic bacteria in each gram of the final product/yogurt, subsequent inoculation was performed with 0.05 g of each culture (lyophilized powder form) per kilogram of milk. Then, the probiotic samples were incubated at 42°C until the pH reached 4.6. The yogurt sample without CSPE was prepared and considered as the control. The yogurt samples were kept in a refrigerator and analyzed for textural characteristics (hardness, consistency, and viscosity index) and total acceptance during 1, 11, and 21 days of storage period. The yogurt microstructure was also evaluated after 21 days' storage at the refrigerator.

### 2.4 Texture analysis test

Textural characteristics of the functional yogurt samples were measured according to the method of Jooyandeh et al. (2015) with some modifications. The texture parameters of samples were evaluated by a texture analyzer (TA.XT.PLUS, Micro stable system, UK) equipped with a load cell of 5 kg. The cylindrical probe (36 mm) constantly pressed the yogurt samples up to 50% of its initial height. Pretest, test, and posttest speeds were adjusted to 1 mm/s with a trigger system using a force of 5 g. The texture parameters including firmness (maximum force [mg]), consistency (area within curve throughout extrusion plunge [mg/s]), and index of viscosity (negative area of curve meanwhile probe removal [mg/s]) were measured.

### 2.5 Enumeration of probiotic bacteria

The count of *L. acidophilus LA5* was evaluated through the serial dilution technique and using the pour plate technique. One‐milliliter aliquots from each yogurt sample were successively diluted with peptone water (0.1%) and 10^−5^–10^−7^ dilutions were cultured on MRS agar containing 0.15% bile salts (Torabi et al., 2021). After incubation at 37°C for 72 h, the viability of probiotics in the plates (with 25–250 colonies) was recorded and expressed in log cfu/g.

### 2.6 Microstructural test

Microstructure of the set yogurts was evaluated by scanning electron microscopy (SEM) technique according to Danesh et al. (2018) with some modifications. In order to microstructural assessment, yogurt samples were splintered with a blade and the segments were fixed on aluminum SEM stumps. Then, samples were cryo‐fractured by dipping in liquid nitrogen and coated with gold. The scanning electron micrographs were carried out through a SEM (TESCAN, VEGA model, Czech Republic) adjusted at 10.0 KV. SEM photomicrographs were taken at 500–5000× magnifications.

### 2.7 Sensory evaluation

Sensory evaluation is a significant division of dairy product advancement and production, usually based on hedonic consumer responses amongst others (Cheng et al., 2022). Simple and adjustable developing sensory methods have been suggested to achieve rapid results at a lesser price. However, among the sensory procedures used for dairy products, conventional descriptive analysis has been continually performed and is well founded (Ribeiro et al., 2024). The overall acceptance of the fortified yogurt samples was judged by 20 semitrained panelists (students and faculty members of the university) according to the ISO method (ISO, 2023). The overall acceptance of probiotic yogurts was assessed using 9‐point hedonic scale (where 1 meant “dislike extremely” and 9 “like extremely”) and based on the sensory attributes (including odor, color, texture, and taste).

### 2.8 Statistical analysis

In the present study, yogurt samples were fortified with 5% CSPE obtained from three different colors of sweet peppers. Yogurt sample (without CSPE) is regarded as control. Four fortified yogurts were manufactured and the textural characteristics and product acceptability of the samples were assessed during 21 days of storage (in 10‐day intervals). A completely randomized factorial design (4 × 3) was utilized to evaluate the main and interaction effects of the factors on the selected responses. One‐way analysis was also done to govern substantial variances between yogurt treatments through the storage time. SPSS software (version 20) was used for the statistical analysis of the parameters and the means of evaluated parameters were compared with Duncan's test at 95% confidence level.

## 3 RESULTS AND DISCUSSION

### 3.1 Effect of CSPE on texture characteristics

#### 3.1.1 Hardness

**FIGURE 1:**
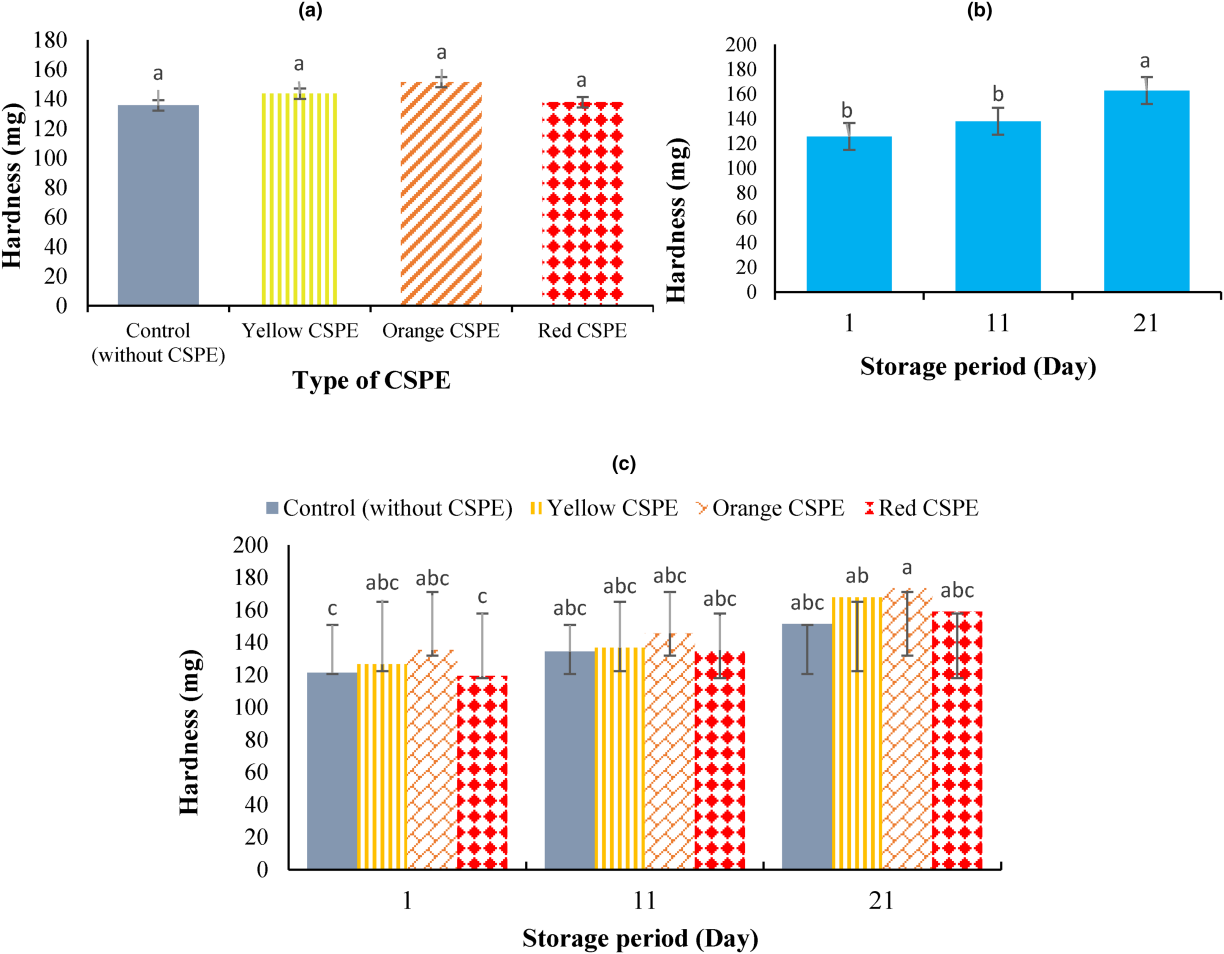
Effect of the addition of 5% concentrated sweet pepper extract (CSPE) (a), storage period (b), and their interaction (c) on hardness of the set yogurt samples.

Textural properties of yogurt have a substantial impact on its sensory perception and ultimately acceptance of the product by final consumer (Prajapati et al., 2016). Set yogurt is a viscoelastic matter whose elastic behavior mostly described by its firmness, consistency, and viscosity. Results showed that the incorporation of concentrated CSPE in the fortified yogurts had no effect on the yogurt hardness, but the storage time and the interaction of CSPE×storage time had important influences on this parameter (Figure 1).

As shown in Figure 1a, with the incorporation of CSPE in the yogurt samples, the yogurt firmness did not change significantly. Although fortified yogurt samples (having 5% CSPE with 30% T.S.) had higher total solids than control, yogurt hardness did not affect (*p* > .05). This is probably owing to the destabilization of 3D gel network by the addition of CSPE as a result of its interference with casein micelles and altering casein interactions (Jooyandeh et al., 2017; Paseephol et al., 2008). In a similar result, Yang et al. (2012) stated that the addition of ginger juice led to a significant reduction of yogurt firmness, likely because of the inhibition of lactic acid bacteria causing incomplete acid gel of casein. Bulut et al. (2021) also stated that fortification of set yogurts with different plant extracts had significant effects on yogurt firmness during 21 days of cold storage. They reported that the addition of mint, grape seed, green tea, and rhubarb extracts caused significant increase in the yogurt firmness while the addition of thyme extract had adverse effect and substantially reduced the yogurt firmness.

As seen in Figure 1b, as the storage time increased, the amounts of firmness increased meaningfully. The amount of hardness on the first day of storage was 125.67 g and it increased to 138 and 162.83 g after 11 and 21 days of cold storage, respectively (Figure 1b). Among yogurt samples, yogurt sample having orange CSPE at the end of storage with 173.33 g had the highest firmness, and sample having red CSPE at the initial storage period with 119.33 g had the lowest firmness (Figure 1c). The increase in yogurt firmness during the storage period has been associated with casein network rearrangement and improvement of protein linkages (Yademellat et al., 2017). Furthermore, in all the storage intervals, no substantial differences were observed between fortified treatments and control. Such effects have also been reported in earlier research, in which the plant extracts could alter the yogurt structure, independent of the storage period, owing to the dispersion of casein micelles (Domagala et al., 2005; El‐Sayed & Youssef, 2019). According to our results, Nikbakht Kashkouli et al. (2017) reported a constant increase in yogurt (dahi) firmness over a 19‐day storage period.

#### 3.1.2 Consistency

**FIGURE 2:**
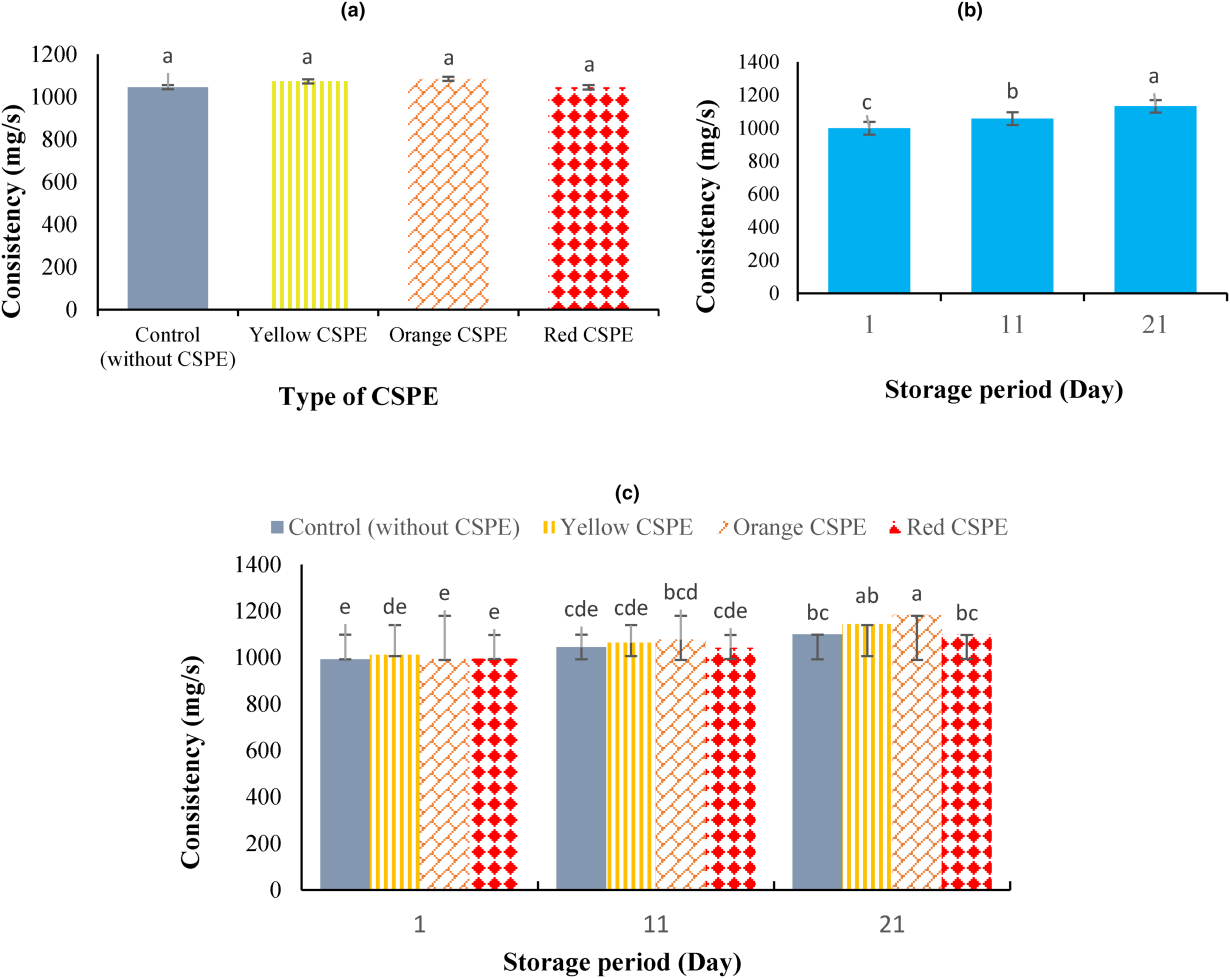
Effect of the addition of 5% concentrated sweet pepper extract (CSPE) (a), storage time (b), and their interaction (c) on consistency of the set yogurt samples.

Consistency is an important criterion for the consumer acceptance of dairy products including yogurt. The consistency of yogurt samples (area under the curve during penetration) was calculated for each of the four treatments during 21 days of cold storage (Figure 2). As shown in Figure 2a, similar to firmness, CSPE fortification did not have a significant effect (*p* > .05) on yogurt consistency, whereas significant changes (*p* < .05) were observed over the cold storage period. The amount of consistency for control and fortified yogurt samples having yellow, orange, and red CSPEs was determined as 1045.33, 1073.33, 1084.56, and 1045.22 g/s, respectively. Yogurt gels are prominently affected by the total solid content of the yogurt milk, particularly the protein content. Viscosity and consistency of yogurt increase with rise in total solid content of milk (Prajapati et al., 2016). However, despite higher total solids, fortified yogurt samples did not vary in terms of consistency as compared with control due to the negative effect of CSPE on casein network.

Results showed that over 21 days of storage period, the initial amount of yogurt consistency increased from 998.25 g/s to 1131.25 g/s at the end of storage period (Figure 2b). The increase in the amount of consistency during the cold storage may be due to the proteolysis (rises in the polarity groups) and subsequently increase in water holding capacity (Jooyandeh & Minhas, 2009; Torabi et al., 2021) and/or could be associated with the evaporation of water throughout the storage (Abdeldaiem et al., 2023). Among yogurt samples during the storage period, yogurt sample having orange CSPE at the end of storage with 1183.33 g/s had the highest consistency (Figure 2c). However, there was no major variance between this sample and yogurt having yellow CSPE at the end of 21 days of cold storage. Furthermore, no important changes in yogurt consistency between control and fortified samples were observed at the initial and middle of the storage period. The higher changes in yogurt consistency in the fortified samples as compared to control during the storage are probably due to the presence of dietary fiber present in CSPE. Dietary fiber is capable of absorbing more free water due to its higher water‐holding capacity (Hashim et al., 2009) and, therefore, prevents yogurt syneresis and increases the consistency of the product.

#### 3.1.3 Viscosity index

The index of viscosity or thickness refers to the resistance of sample to gradual deformation against shear stress, that is, how the viscosity of fluid changes with stress. Analysis results obtained from main effects of the independent variables and their interactions showed that by increasing the CSPE concentration and the storage time, the index of viscosity increased but these differences were not significant (results are not shown). The improvement of yogurt viscosity by adding CSPE is probably owing to the correlation between CSPE compounds, including proteins and phenolic compounds with yogurt proteins, leading to the development of a firmer 3D network gel structure (Mohamed Ahmed et al., 2021). The results were similar to those described by Yademellat et al. (2017) who reported that the addition of Persian and Balangu‐Shirazi gums and time of storage had no effect on the index of viscosity. Contrary to our result, Wang et al. (2020) even showed that the addition of apple pomace to a diluted yogurt system cause a significant increase in viscosity index and has latent to stabilize the yogurt drink and decrease the precipitation of coagulated protein particles, whereas Mohammadi‐Gouraji et al. (2019) found that the viscosity of yogurts enriched with phycocyanin powder was inferior to plain yogurt. This is mainly due to microstructure alterations and disruption/breakdown of yogurt gel, which result in a lower surface tension and inferior viscosity.

### 3.2 Counting of probiotic bacteria

**FIGURE 3:**
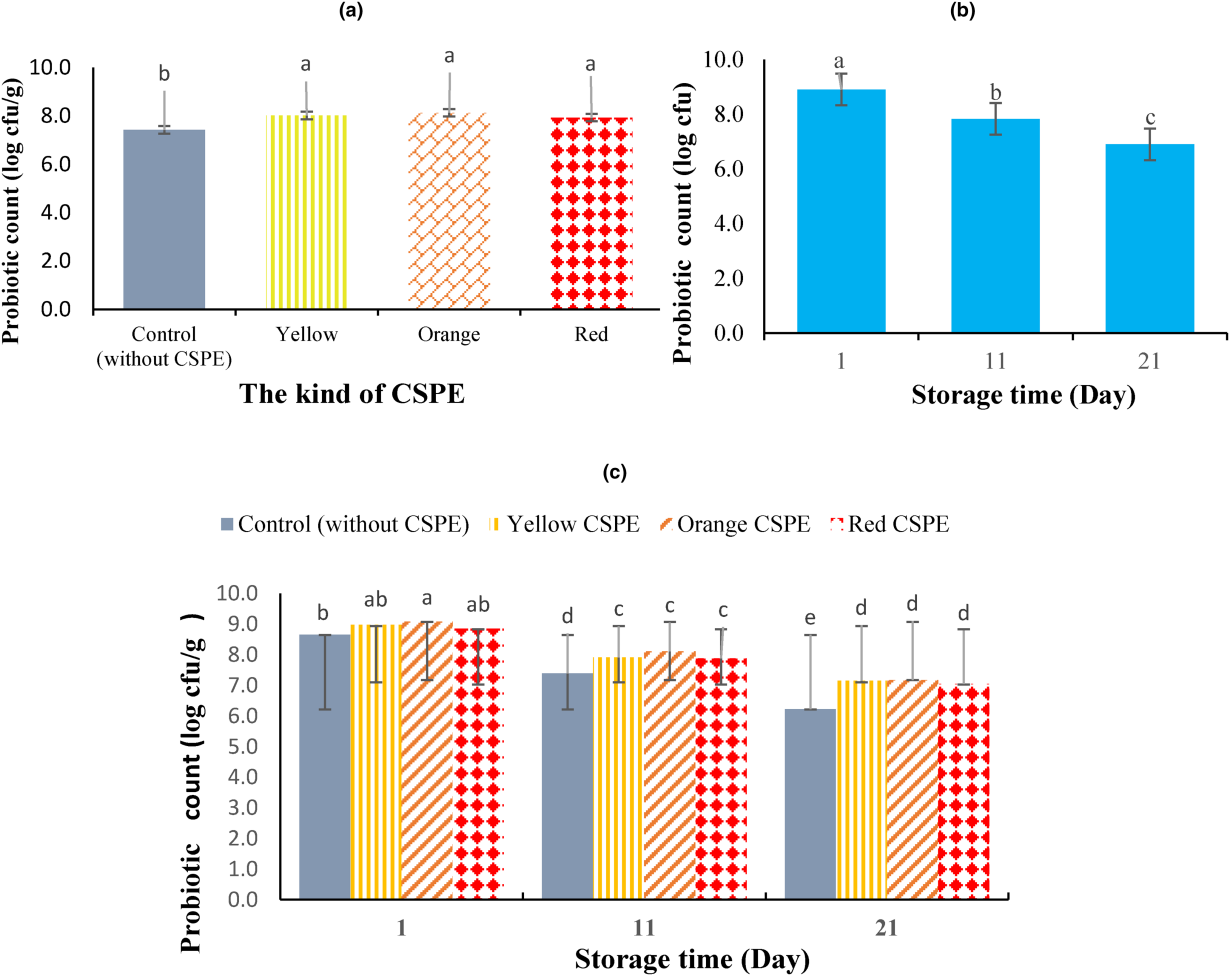
Effect of the addition of 5% concentrated sweet pepper extract (CSPE) (a), storage time (b), and their interaction (c) on probiotic count of the set yogurt samples.

Figure 3 shows the numbers of the survived strain of *L. acidophilus* LA‐5 in probiotic yogurts throughout 21 days of storage period. The count of probiotic bacteria meaningfully (*p* < .01) enhanced with incorporation of CSPE (Figure 3a). Adding 5% CSPE caused a high growth increment of probiotic bacteria in fortified yogurts containing yellow, orange, and red CSPE (8.02, 8.13, and 7.93 log cfu/g, respectively) as compared to the yogurt control (7.42 log cfu/g). The upsurge in the count of probiotic bacteria in fortified samples may be indorsed with the prebiotic effect of CSPE constituents. Prebiotics promote survival of probiotics by motivating their metabolism and growth, supplying vital nutrients, and altering aggressive surrounding area statuses (Lourens‐Hattingh & Viljoen, 2001). It is shown that phenolic substances stimulate the proliferation of probiotics (Shirani et al., 2022). Moreover, dietary fibers and proteins survive these beneficial microorganisms in acidic conditions (Senadeera et al., 2018). It is reported that by adding antioxidant substances such as vitamin C, the number of *Bifidobacterium animalis* and *B. infantis* bacteria increased by 150%–190% (Xin‐Huai & Dan, 2009). In similar results, Jeong et al. (2018) reported a higher number of probiotics as a consequence of adding 1%–3% of green tea powder to yogurt. Amirdivani and Baba (2015) also showed that the count of probiotic Lactobacillus bacteria in yogurt having green tea extract was almost twice as much as the control yogurt owing to the incidence of phenolic compounds.

The results also showed that storage time caused a significant difference in the count of probiotic bacteria. According to Figure 3b, the number of probiotic bacteria decreased significantly throughout the storage time. The decrease in the number of *L. acidophilus* LA‐5 throughout the cold storage period in yogurt and other dairy products has been reported by many researchers (Jooyandeh et al., 2023; Kang et al., 2018; Torabi et al., 2021) and this decrease is mainly attributed to the reduction of milk compounds and the increase of acidity.

With a brief view at Figure 3c, it can be seen that the number of *L. acidophilus* LA‐5 in the control yogurt (6.23 log cfu/g) at the end of 21 days of storage is significantly lower than the yogurt sample containing CSPE (7.05–7.18 log cfu/g). In agreement with our results, Zahid et al. (2022) stated that the number of probiotic bacteria in yogurt samples containing mango and banana peel powder was much higher than in control yogurt.

### 3.3 Microstructure evaluation

**FIGURE 4:**
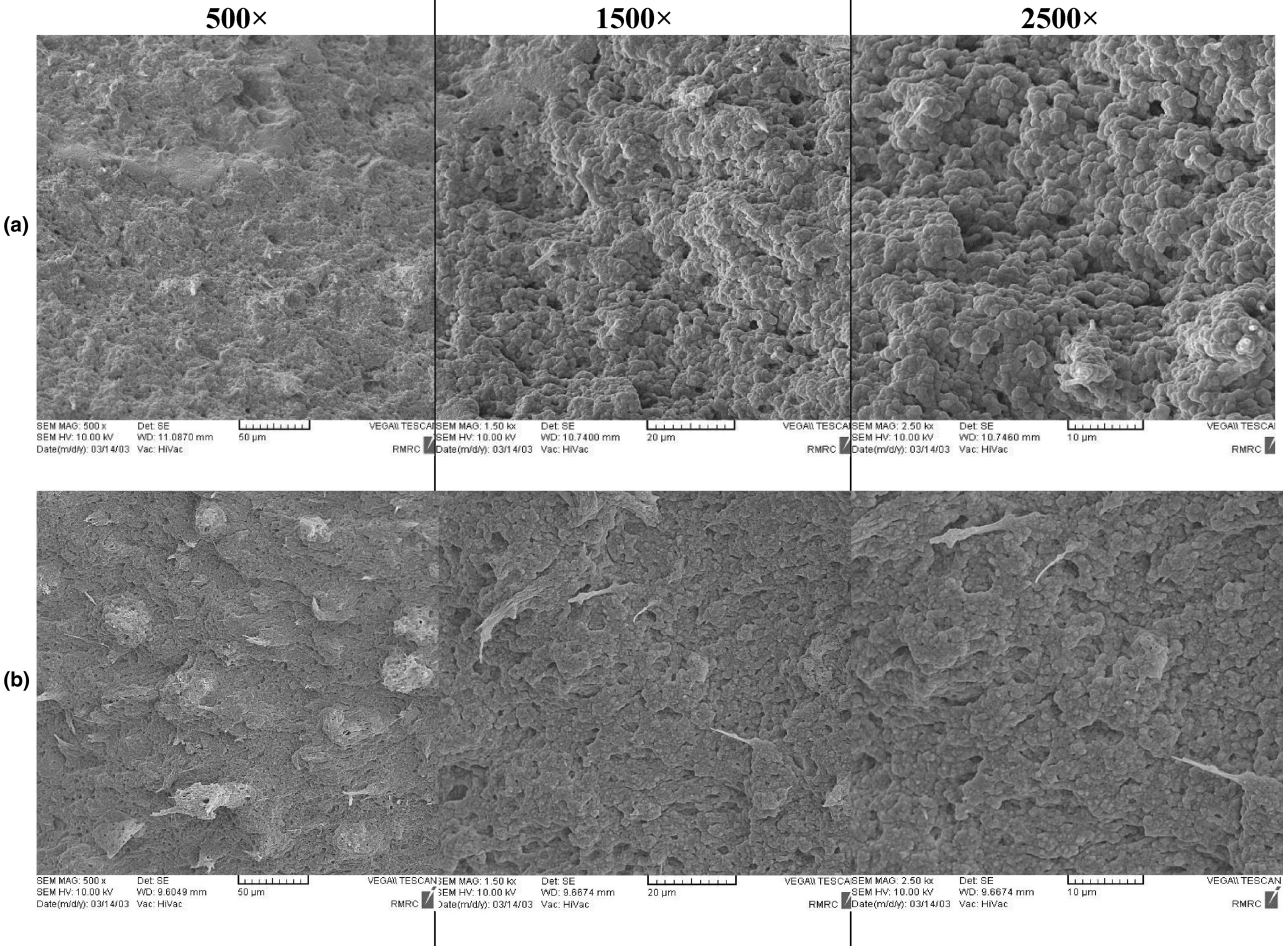
Scanning electron microscopy images of control yogurt (without concentrated sweet pepper extract [CSPE], a) and fortified orange CSPE yogurt (b) at the end of 21 days of cold storage period (with magnification of 500×, 1000×, and 2500×).

Scanning electron microscopy images of control yogurt (without CSPE) and fortified orange CSPE yogurt are presented in Figure 4. Results showed that the addition of CSPE had undoubtedly a mutual effect on yogurt microstructure. Fortified CSPE yogurt (Figure 4b) displayed large flakes with irregular surface and more compact texture as compared to the control sample (Figure 4a). The flakes and uneven surface of CSPE yogurt are probably caused by destabilization of 3D casein network due to the interference of CSPE compounds with casein micelles (Jooyandeh et al., 2017; Paseephol et al., 2008). However, interaction between some CSPE compounds, such as phenolic compounds with yogurt proteins (Mohamed Ahmed et al., 2021), likely resulted in the development of a firmer 3D network gel structure which led to a condensed texture. Therefore, as mentioned before, insignificant differences were found between fortified and plain yogurt in terms of textural properties. Similarly, Qin et al. (2023) reported that yogurt fortification with high‐quality dietary fiber attained from the by‐products of grapefruit caused a compact structure with a little wrinkled surface. Ibrahim et al. (2020) also reported that the addition of pomegranate peel to low‐fat bio‐yogurt improved the final product's smoothness and viscosity.

### 3.4 Sensory evaluation

Consumer acceptance of yogurt is based on its physical aspects like textural properties (particularly the amount of hardness and perceived consistency), color insights, odor perceptions, and taste (particularly acidity). Enrichment of yogurt with plant extracts having bioactive‐rich substances is one of the various approaches to overcome the diseases related with nutrient shortages (Bulut et al., 2021). However, this strategy may change product quality, particularly sensory attributes. The changes in overall acceptability of the yogurt treatments during cold storage period are indicated in Table 1. The control sample (yogurt without CSPE) had considerably higher acceptance score (8.12) than yellow (7.58), orange (7.66), and red (7.56) CSPE samples. However, all the CSPE‐containing yogurts had an acceptable score (<7 or higher than good) in all storage periods (Table 1). In agreement with our results, Kang et al. (2018) reported a decrease of more than 50% in the overall acceptance of stirred yogurt as a result of adding 5% fermented extract of red and green pepper. Hashim et al. (2009) also reported that yogurt fortified with 3% date fiber had similar total acceptability score as compared with control yogurt, and increasing date fiber to 4.5% caused a significant decrease in total acceptability. On the contrary, Khalifa and Gomaa (2021) reported an increase in overall acceptance score in yogurt drinks containing plant extracts from the dried roselle calyces, peanut skins, and outer peels of yellow onions.

**TABLE 1:** Effect of the addition of 5% concentrated sweet pepper extract (CSPE) with different colors on total acceptability of the set yogurt samples during 21 days of storage at 4°C.

Characteristics	Storage time (day)	Bell pepper extract (CSPE)			
		Control (without CSPE)	Yellow	Orange	Red
Overall acceptability	1	8.31 ± 0.22^aA^	8.10 ± 0.12^aA^	8.12 ± 0.22^aA^	8.15 ± 0.23^aA^
	11	8.20 ± 0.15^aAB^	7.54 ± 0.12^bB^	7.71 ± 0.35^bA^	7.49 ± 0.25^bB^
	21	7.86 ± 0.16^aB^	7.11 ± 0.33^bC^	7.16 ± 0.12^bB^	7.05 ± 0.16^bC^

*Note*: Different small and capital letters indicate significant differences (*p* < .05) in each row and column, respectively.

In addition to the effect of adding CSPE, the storage period also led to a momentous decrease in the overall acceptance score of the samples. The average overall acceptance score of the yogurt samples at the beginning of the storage time was determined as 8.17, which decreased to 7.74 in the middle of storage and to 7.29 at the end of 21 days of cold storage. A decrease in the sensory score during storage has also been reported by other researchers (Khalifa & Gomaa, 2021; Salah et al., 2022). Mousavi et al. (2019) also stated that the total acceptance of flaxseed‐enriched yogurt was meaningfully (*p* < .01) decreased by enhancing flaxseed concentration and storage time. They suggested that the sensory characteristic of flaxseed‐enriched yogurt may be improved by the addition of a flavoring agent.

According to Table 1, the highest overall acceptance with a score of 8.31 was related to the control sample on the first day of the storage period, and the lowest with a score of 7.05 was related to the yogurt sample containing red CSPE on the 21st day of storage. The results showed that although at the initial storage time, there was no difference between the overall acceptance scores of yogurt samples containing CSPE and the control sample, with the passage of storage time, these differences became significant (Table 1). The greater decrease in the sensory score of samples containing CSPE compared to the control yogurt in the middle and end of the storage periods can be due to significant changes in the taste and color of yogurt due to the oxidation of CSPE compounds such as carotenoid pigments (Oliveira et al., 2015; Wallace & Giusti, 2008).

## 4 CONCLUSION

In this investigation, we studied the effect of the addition of concentrated extracts of yellow, orange, and red sweet pepper on some quality parameters of functional set yogurt throughout storage. Our results showed that the yogurt texture could be maintained or even upgraded by using natural fortificant substances. Incorporation of CSPE increased survivability of the probiotic bacteria but it had a negative effect on the total acceptability of the set yogurts. Nevertheless, all the fortified yogurt samples had acceptance scores of more than 7 (i.e., good acceptability) during the storage period. By passing the storage time, the amounts of hardness and consistency increased, while the amounts of total acceptability and the count of probiotics decreased meaningfully (*p* < .01). In conclusion, based on textural parameters, viable probiotic bacteria count, and total acceptability, the yogurt sample having orange CSPE was selected as the best functional product. At the end of 21 days of storage, the probiotic count of this fortified yogurt (6.23 log cfu/g) was significantly higher than control (7.18 log cfu/g). By consuming this plant‐fortified yogurt, in addition to receiving considerable amount of bioactive substances, the regular recommended levels of probiotic bacteria could be attained.

## AUTHOR CONTRIBUTIONS

**Hossein Jooyandeh:** Conceptualization (lead); data curation (lead); formal analysis (lead); funding acquisition (lead); investigation (equal); methodology (equal); project administration (lead); resources (equal); validation (equal); writing – original draft (lead); writing – review and editing (lead). **Behrooz Alizadeh Behbahani:** Investigation (equal); methodology (equal); resources (equal); validation (equal).

## CONFLICT OF INTEREST STATEMENT

The authors have declared no conflict of interest.

## ETHICS STATEMENT

This study does not involve any human or animal testing.

## INFORMED CONSENT

Written informed consent was obtained from all study participants. The manuscript is not submitted or under consideration in any other journal.

## Data Availability

Data will be made available on request.
